# Universal and reusable virus deactivation system for respiratory protection

**DOI:** 10.1038/srep39956

**Published:** 2017-01-04

**Authors:** Fu-Shi Quan, Ilaria Rubino, Su-Hwa Lee, Brendan Koch, Hyo-Jick Choi

**Affiliations:** 1 Department of Medical Zoology, Kyung Hee University School of Medicine, Seoul, 130-701, Korea; 2 Department of Chemical and Materials Engineering, University of Alberta, Edmonton, AB T6G 1H9, Canada; 3 Department of Biomedical Science, Graduate School, Kyung Hee University, Seoul, 130-701, Korea

## Abstract

Aerosolized pathogens are a leading cause of respiratory infection and transmission. Currently used protective measures pose potential risk of primary/secondary infection and transmission. Here, we report the development of a universal, reusable virus deactivation system by functionalization of the main fibrous filtration unit of surgical mask with sodium chloride salt. The salt coating on the fiber surface dissolves upon exposure to virus aerosols and recrystallizes during drying, destroying the pathogens. When tested with tightly sealed sides, salt-coated filters showed remarkably higher filtration efficiency than conventional mask filtration layer, and 100% survival rate was observed in mice infected with virus penetrated through salt-coated filters. Viruses captured on salt-coated filters exhibited rapid infectivity loss compared to gradual decrease on bare filters. Salt-coated filters proved highly effective in deactivating influenza viruses regardless of subtypes and following storage in harsh environmental conditions. Our results can be applied in obtaining a broad-spectrum, airborne pathogen prevention device in preparation for epidemic and pandemic of respiratory diseases.

Aerosols take a prominent role in airborne transmission of respiratory diseases. Droplets with aerodynamic size (d_a_) < 10 μm and 10 < d_a_ < 100 μm are known to infect the alveolar regions and upper respiratory tract, respectively^1^,^2^. Notably, aerosols can also be a route of infection in diseases that, contrary to for instance influenza, do not specifically target the respiratory tract, as it could be the case of Ebola virus^3^. While vaccination can greatly reduce morbidity and mortality, during a pandemic or epidemic new vaccines matching the specific strain would be available, at the earliest, six months after the initial outbreak. Additionally, following development of an effective viral vaccine, several potential problems would remain, such as limited supply due to insufficient production capacity and time-consuming manufacturing processes. As a result, individuals close to the point of an outbreak would be in imminent danger of exposure to infectious diseases during the non-vaccine period. In the absence of vaccination, respirators and masks can be worn to prevent transmission of airborne pathogenic aerosols and control diseases, such as influenza^4^.

The main alternative, the N95 respirator, requires training prior to use, must be expertly fitted to address the risk of faceseal leakage at the face-mask interface, and must be disposed of as biohazard^5^. Due to these factors, the use of N95 respirators on a large scale is impractical and expensive during an epidemic or pandemic. Past experiences of severe acute respiratory syndrome (SARS), H1N1 swine flu in 2009, and Middle East respiratory syndrome (MERS) indicate that surgical masks have been most widely adopted by the public as personal protective measure, despite controversy on their effectiveness^6^,^7^,^8^,^9^. Currently, among other factors, filtration in respirators and masks depends on filter characteristics, including fiber diameter, packing density, charge of fibers and filter thickness, as well as particle properties, such as diameter, density and velocity^10^,^11^,^12^,^13^,^14^. However, in the lack of a system to deactivate the collected pathogens, safety concerns naturally arise about secondary infection and contamination from virus-laden filter media during utilization and disposal. Furthermore, since re-sterilization is not possible without causing damage, respirators and masks are recommended for single use only^9^,^15^,^16^. Scientific efforts have been focused on treatment of filters with materials possessing well-known antimicrobial properties, such as iodine, chlorine and metals^17^,^18^,^19^,^20^,^21^,^22^,^23^,^24^,^25^, although with limited effectiveness against virus aerosols^26^,^27^,^28^. Therefore, a key challenge is the development of an easy-to-use, universal virus negation system, which is reusable without reprocessing and capable of deactivating pathogens, thereby reducing potential risk of secondary infection and transmission.

Here, we report a simple but efficient virus inactivation system exploiting the naturally occurring salt recrystallization. Our strategy is to modify the surface of the fibrous filtration layer within masks with a continuous salt film for virus deactivation via two successive processes: i) salt is locally dissolved by the viral aerosols and ii) supersaturation is followed by evaporation-induced salt recrystallization. Consequently, viruses are exposed to increasingly higher concentrations of saline solution during drying and physically damaged by recrystallization.

## Results

### Preparation and characterization of salt-functionalized filters

**Figure 1:**
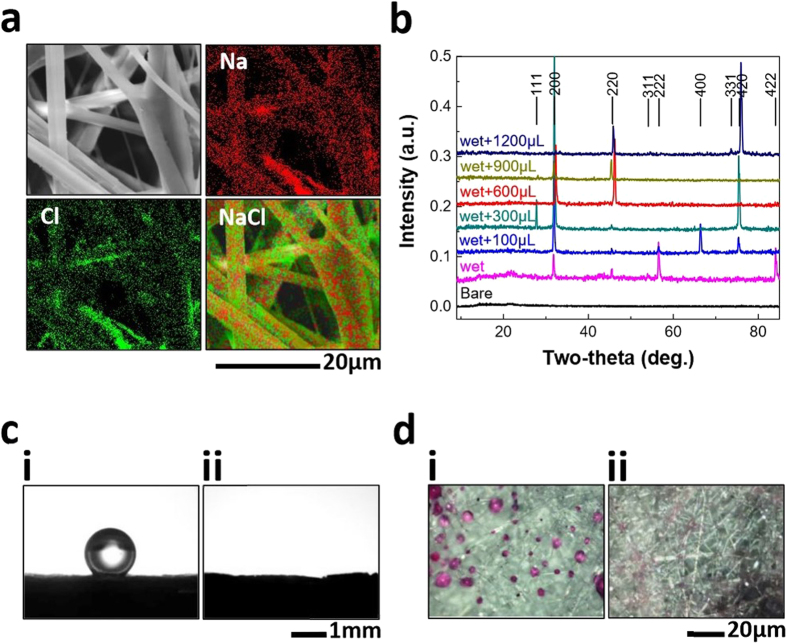
Mask with salt-coated filter for prevention and deactivation of airborne pathogens. (**a**) SEM image of Filter_wet+600μL_ (top left) and EDX mapping images of Na (red), Cl (green), and NaCl (combination of Na and Cl mapping images), showing the formation of NaCl coating, as also confirmed by XRD spectra (**b**) of Filter_bare_, Filter_wet_, Filter_wet+100μL_, Filter_wet+300μL_, Filter_wet+600μL_, Filter_wet+900μL_ and Filter_wet+1200μL_ (labelled as Bare, wet, wet+100 μL, wet+300 μL, wet+600 μL, wet+900 μL and wet+1200 μL, respectively; miller indices corresponding to NaCl crystal are shown at the top of XRD spectra for each position). (**c**) Optical microscope images for contact angle measurements using 3 μL DI water droplets on (i) Filter_bare_ and (ii) Filter_wet+600μL_ (*n* = 10). (**d**) Microscope images of aerosol on (i) Filter_bare_ and (ii) Filter_wet+600μL_ (*n* = 10).

To demonstrate the concept of virus deactivation system based on salt recrystallization, the middle layer of three-ply surgical mask, polypropylene (PP) microfiber filter, was coated with NaCl salt as an active virus negation unit (see Supplementary Fig. S1 for bare PP filter). The coating formulations contained surfactant to enhance wetting of saline solution on the surface of hydrophobic PP fibers. Bare PP filters (abbreviated as Filter_bare_) were pre-wet to contain about 600 μL of coating solution (abbreviated as Filter_wet_). The amount of NaCl salt (W_salt_ in mg/cm^2^) coated on the filter per unit area, considering that the filters thickness is constant, was easily controlled by changing the coating solution volume (V_salt_ in μL) during drying of pre-wet filter (radius: 3 cm, W_salt_ = 3.011 + 0.013 × V_sat_, *n* = 7) (Fig. S2). Scanning electron microscopy (SEM) and energy dispersive X-ray (EDX) mapping analysis showed the formation of homogeneous NaCl coating during drying, as also confirmed by X-ray diffraction (XRD) (Fig. 1a,b and Supplementary Fig. S3). Both the formation of NaCl coating on PP fibers and presence of surfactant in the coating formulation appeared to alter the filter surface properties from hydrophobic (bare filter; contact angle, θ_c_ = 133.0 ± 4.7°) to completely hydrophilic (salt-coated filter; θ_c_ ~ 0°, *n* = 10) (Fig. 1c and Supplementary Fig. S4). Hydrophilic nature of salt coating can greatly improve adhesion of viral aerosols to PP fibers compared to Filter_bare_, as seen in Raman microscope images (Fig. 1d and Supplementary Fig. S5).

### Filtration efficiency against viral aerosols and protective efficacy *in vivo*

**Figure 2:**
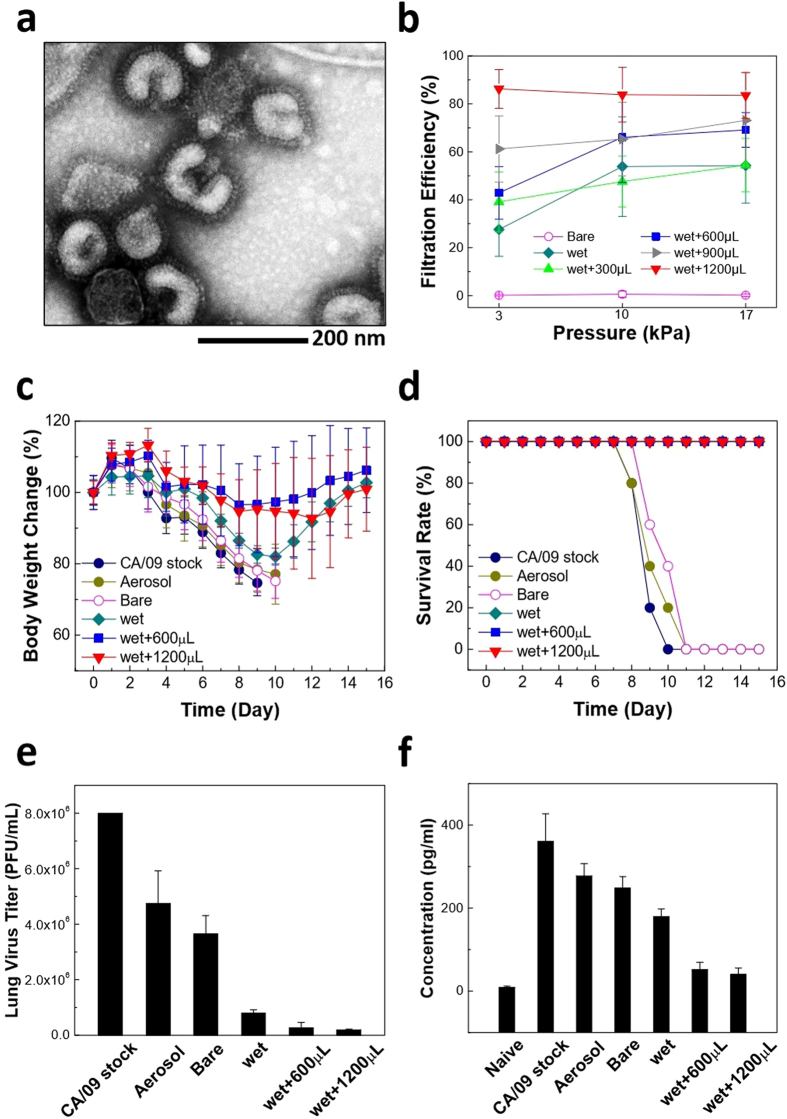
Filtration efficiency of salt-coated filters. (**a**) TEM image of CA/09 H1N1 influenza virus. (**b**) Pressure-dependent filtration efficiency (*n* = 8–10, mean ± standard deviation (SD)). (**c**–**f**) Effects of filtration efficiency on protective efficacy *in vivo*. Body weight change of mice after infection with the dosages of penetrated virus (*n* = 12, mean ± SD) (**c**), survival rates (mean; 100% means that all mice in the group survived as penetrated dosages were lower than lethal dose) (**d**), lung virus titers (*n* = 4, mean ± SD) (**e**), and lung inflammatory cytokine (interferon-γ (IFN-γ)) assay (*n* = 11, mean ± SD) (**f**). Legends: filters are labelled as in Fig. 1b.

Filtration efficiency of salt-coated filters was tested against aerosols with volumetric mean diameter (VMD) of 2.5–4 μm containing H1N1 pandemic influenza virus (A/California/04/2009, abbreviated as CA/09) at different pressure conditions (see Fig. 2a for transmission electron microscope (TEM) image of H1N1 virus). Interestingly, as shown in Fig. 2b, Filter_bare_ did not exhibit any significant level of resistance against penetration of virus under our experimental conditions (i.e., 0% filtration efficiency). Conversely, salt-coated filters showed substantially increasing filtration efficiency with pressure and amount of coated salt. In particular, in the case of Filter_wet+600μL_, filtration efficiency varied from 43 to 70%, with increasing pressure from 3 to 17 kPa, and Filter_wet+1200μL_ exhibited persistent, high-level efficiency (~85%) (one-way ANOVA, *P* = 0.85).

To probe the effects of filtration efficiency on protective efficacy, *in vivo* experiments were performed using mice intranasally (IN) infected with penetrated dosages of H1N1 virus under breathing pressure (~10 kPa)^29^. As shown in Fig. 2c, similarly to negative control groups (mice infected with lethal dose of virus stock and aerosolized virus), mice exposed to a dose penetrated through the bare filter showed rapid body weight loss, followed by death within 10 days after infection, in good agreement with the observed 0% filtration efficiency (Fig. 2b). In contrast, mice groups exposed to virus derived from salt-coated filters resulted in 100% survival rate (Fig. 2d). Furthermore, lungs of mice from negative control groups exhibited severe lung infection 4 days after challenge (Fig. 2e). Conversely, mice groups exposed to virus derived from salt-coated filters showed significantly lower levels of lung viral titers (t-test, *P* < 0.005). This is consistent with lower levels of inflammatory cytokines, interferon-γ (IFN-γ), from salt-coated filter groups compared to negative control and bare filter groups (t-test, *P* < 0.001) (Fig. 2f).

### Deactivation of virus on salt-functionalized filters

**Figure 3:**
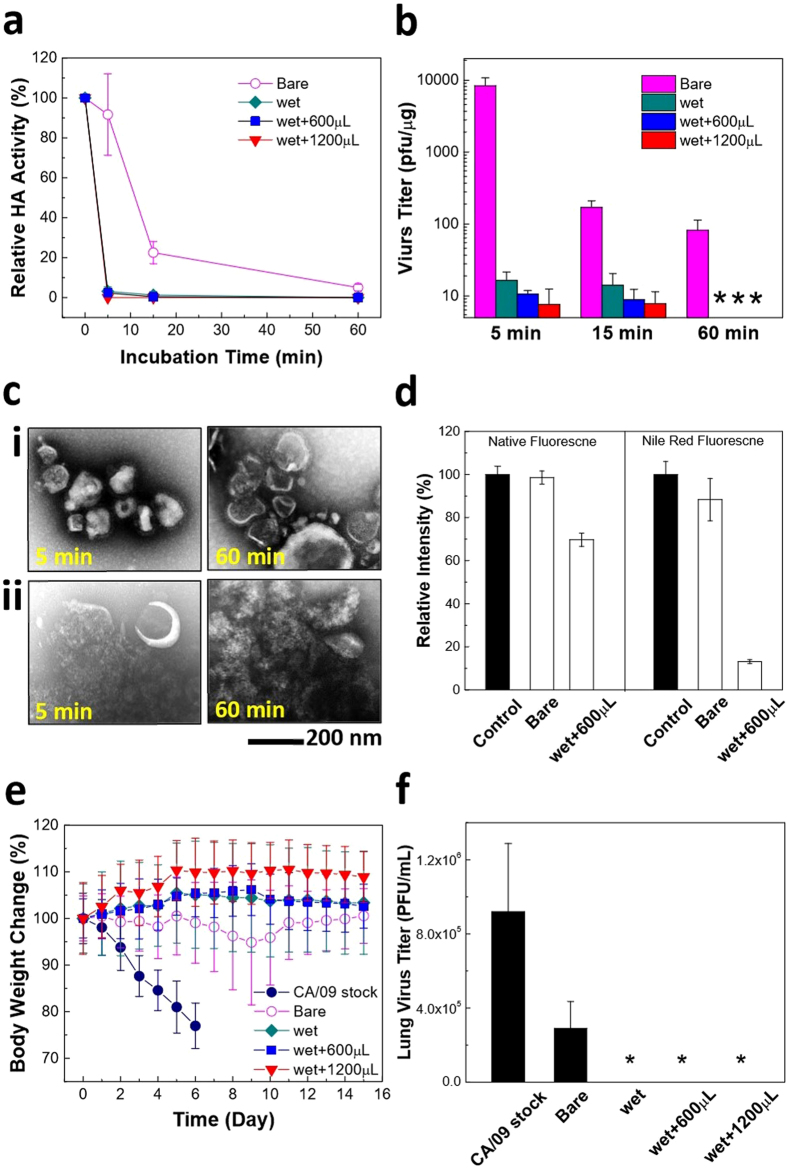
Inactivation of virus adsorbed on salt-coated filters. (**a**,**b**) HA activity (**a**) and virus titer (**b**) displaying the effects of incubation time on the remaining activity of virus (*n* = 4–8, mean ± SD). (**c**) TEM images of viruses reconstituted, after incubation for 5 and 60 min, from (i) Filter_bare_ and (ii) Filter_wet+600μL_. (**d**) Native fluorescence/nile red fluorescence of viruses incubated for 60 min (*n* = 12, mean ± SD). (**e**,**f**) Body weight change of mice after infection with virus recovered from filters after incubation for 60 min (*n* = 12, mean ± SD) (**e**), and lung virus titers (*n* = 6, mean ± SD) (**f**). Asterisk (*): below detection limit. Legends: filters are labelled as in Fig. 1b.

Influenza virus stability tests were performed to investigate the effects of salt coating. The same amount of recovered viruses from the PP fibers was used, and, in the case of bare filters, viral aerosols exposure was conducted in the absence of pressure due to 100% penetration of viral aerosols. Unlike bare filters (Fig. S6a(i)), formation of micron-sized NaCl phase represents a typical feature of salt-coated filters due to recrystallization of NaCl salt, following local dissolution upon aerosols exposure (SEM images in Fig. S6a, ii to iv, and EDX mapping in Fig. S6b). In contrast to 8% HA activity loss of virus adsorbed onto Filter_bare_, salt-coated filters exhibited almost complete HA activity loss within 5 min of incubation (Fig. 3a). Such dramatic virus destabilization on salt-coated filters is further supported by negligible levels of viral titers compared to Filter_bare_ with incubation time (t-test, *P* < 0.001) (Fig. 3b). It is also noted that virus titers exhibited significant decrease with increase of incubation time and amount of coated salt (ANOVA general linear model, *P* < 0.001). TEM analysis showed that influenza virus on Filter_bare_ experiences morphological change into non-spherical shape during aerosol drying (Fig. 3c(i)). Notably, influenza virus was severely damaged on salt-coated filters even at 5 min of incubation (Fig. 3c(ii)). From microscopic analysis, aerosol drying time was about 3 min, indicating that destruction of virus observed at 5 min is associated with drying-induced salt crystallization. Physical damage of virus due to crystallization was similarly reported as a major destabilizing factor of inactivated influenza virus^30^,^31^. Lower levels of native fluorescence and nile red fluorescence from virus recovered from salt-coated filters accounted for more severe conformational change of antigenic proteins and destabilization of viral envelope, respectively, consistent with TEM analysis (t-test, *P* < 0.001) (Fig. 3d). In parallel, we investigated the separate effect of salt concentration increase on virus stability during the aerosol drying process, irrespective of crystal growth. As displayed in Supplementary Fig. S7, the materials collected in suspension from Filter_wet+600μL_ induced visible morphological transformation of the virus (Supplementary Fig. S7b) compared to suspension of Filter_bare_ (Supplementary Fig. S7a). This can be attributed to the high salt/surfactant concentration and osmotic pressure, which have been well-known to destabilize proteins and viruses^31^,^32^,^33^. Therefore, the marked virus destabilization on salt-coated PP fibers can be explained by the combined effects of salt concentration increase during drying and evaporation-induced salt crystallization.

To verify *in vitro* virus stability on the filters, an *in vivo* study was performed by infecting mice with virus incubated for 60 min on PP filters. As shown in Fig. 3e, Filter_bare_ group exhibited 5% body weight loss at day 9 post-infection, reaching a body weight lower than that of salt-coated filter groups by 10–15%. Thus, significantly higher lung virus titers in the negative control group were observed in contrast to no detectable titers in the salt-coated filter groups (Fig. 3f).

### Strain-nonspecific virus deactivation and effects of storage under harsh environmental conditions on salt coating stability

**Figure 4:**
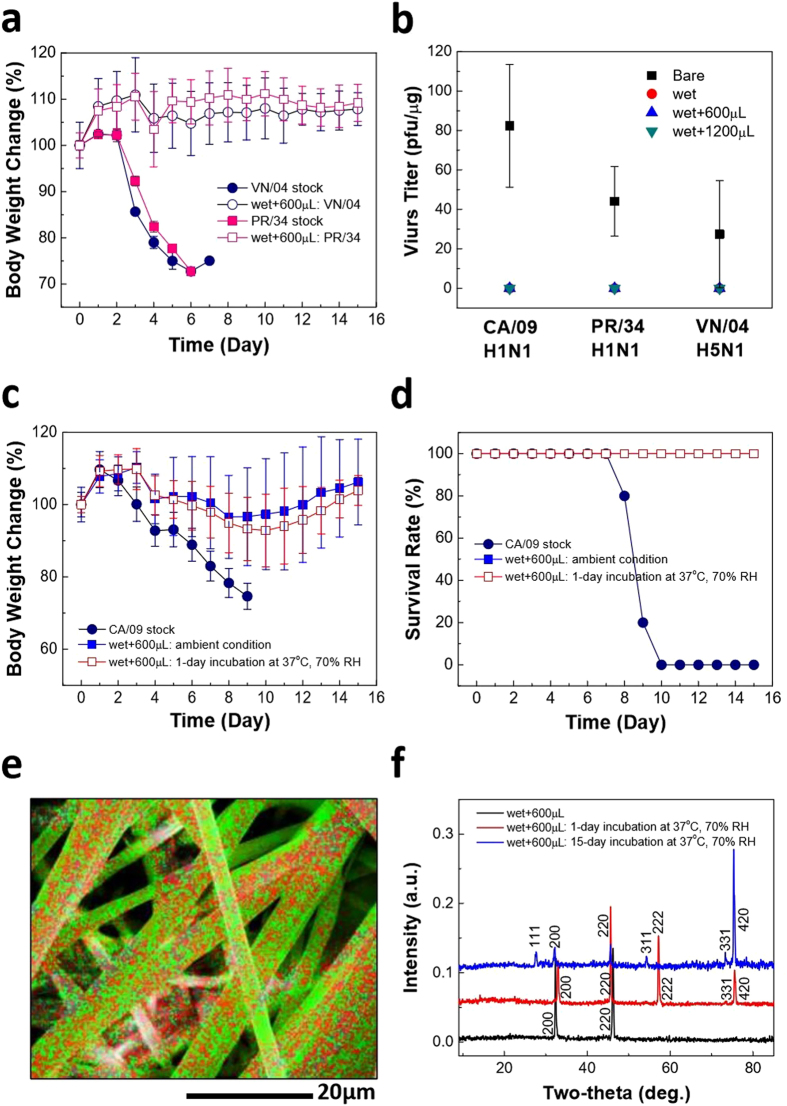
Strain- and environment-dependent performance of salt-coated filters. (**a**) Body weight change of mice infected with penetrated PR/34 H1N1 and VN/04 H5N1 viruses through Filter_wet+600μL_ (*n* = 12, mean ± SD). (**b**) Virus titers of recovered viruses from bare and salt-coated filters (*n* = 4, mean ± SD; data for Filter_wet_, Filter_wet+600μL_ and Filter_wet+1200μL_ are overlapped). (**c**,**d**) Body weight change (**c**) and survival rate (**d**) of mice infected with dosage of penetrated virus through Filter_wet+600μL_ before and after exposure to harsh environmental conditions (37 °C and 70% RH) for 1 day (filled square and open square overlap in (**d**)). (**e**) EDX mapping image of NaCl-coated Filter_wet+600μL_ after incubation for 15 days at 37 °C and 70% RH (combination of Na (red) and Cl (green) mapping images). (**f**) XRD spectra of Filter_wet+600μL_ before and after incubation at 37 °C 70% for 1 day and 15 days. Legends: filters are labelled as in Fig. 1b.

Broad-spectrum protection of salt-coated filters against multiple subtypes of viral aerosols was evaluated by investigating both lethal infectivity by penetrated virus *in vivo* and infectivity by virus collected on filters during filtration *in vitro* using A/Puerto Rico/08/1934 (PR/34 H1N1) and A/Vietnam/1203/2004 (VN/04 H5N1). Similarly to CA/09 H1N1, 100% of mice survived viral infection (PR/34 and VN/04), with no evidence of weight loss, due to higher filtration efficiency of salt-coated filter than that of bare filter (Fig. 4a). This is supported by no significant level of viral titer in the lung. In addition, as shown in Fig. 4b, salt-coated PP filters destroyed adsorbed influenza viruses irrespective of both subtypes and amount of coated salts.

The stability of salt coating on PP fibers was tested under harsh environmental conditions. Incubation at 37 °C and 70% relative humidity (RH) for 1 day did not cause any significant difference in filtration efficiency (t-test, *P* = 0.718) (Supplementary Fig. S8). As a result, all mice infected with dosage of penetrated virus through the filter stored at high temperature and RH displayed 100% survival with 7% of body weight loss (Fig. 4c,d). Even after 15 days of incubation, salts remained to coat PP fibers (Fig. 4e, and Supplementary Fig. S9a,b), despite change in grain orientation due to recrystallization (Fig. 4f, and Supplementary Fig. S10a,b).

## Discussion

Development of a universally applicable, low-cost, and efficient mechanism for virus negation is regarded as a major challenge in public health against general airborne biological threats. This led us to propose a new concept of personal/public preventive and control measures using salt-recrystallization against pathogenic aerosols based on two hypotheses. The salt-coating can enhance adsorption of virus on the filter fibers and inactivate virus by the increase of osmotic pressure followed by the crystallization of salts. As shown in Fig. 2b, salt-coated filters exhibited significantly higher levels of filtration efficiency than bare filters. Notably, the bacterial filtration efficiency (BFE) reported by the mask manufacturer is 99%. The different value of filtration efficiency for bare filters obtained under our experimental conditions may be partially due to the use of aerosols with different biological origins. The FDA-recognized ASTM F2101 – 14 standard for evaluation of BFE exposes surgical masks to *Staphylococcus aureus* aerosols, by employing *S. aureus* ATCC 6538^34^, which has an average diameter of about 1 μm. In this study, filtration efficiency was calculated following exposure of bare and salt-coated filters to influenza virus, which exhibits a smaller diameter than that of *S. aureus* by one order of magnitude. Additionally, whereas during BFE evaluation all three layers of surgical masks are used, in this work filtration efficiency refers to mask filters (middle layer). It is worth noting that the conditions for BFE standard evaluation (such as flow rate and time of application of flow) do not coincide with the experimental procedure we used for measurement of the filtration efficiency, which may further contribute to the different result. The enhanced filtration efficiency of salt-coated filters against influenza virus aerosols as compared to bare filters can be explained by the observed wetting of aerosols, favoring greater adhesion to salt-coated filters. Furthermore, the significant improvement in filtration efficiency resulted in complete protection of mice against lethal influenza aerosols, which demonstrates the high level of protection provided by salt-coated filters, outperforming currently used bare filters.

Rapid loss of HA activity and viral infectivity on salt-coated filters can be explained by physical destruction of virus during recrystallization of coated salts. When the salt-coated filter is exposed to virus aerosols, salt crystals below the aerosol droplet dissolve to increase osmotic pressure to virus. Due to evaporation, the salt concentration of the droplet significantly increases and reaches the solubility limit, leading to recrystallization of salt. As a consequence, virus particles are exposed to increasing osmotic pressure during the drying process and are physically damaged by crystallization. As shown in Fig. 3e,f, the superior advantage of physically destroying the virus adsorbed to the salt-coated PP filters through natural salt crystallization process was further confirmed *in vivo*. According to previous reports, hyperosmotic stress (>541 mOsm) and crystallization induce membrane perturbation with irreversible deformation of the viral envelope and structural virus damage, respectively, resulting in infectivity loss of virus^30^,^31^. Therefore, our data support that the extensive level of infectivity loss associated with a salt recrystallization process caused by physical contact between virus aerosols and salt coating can be used in developing virus negation systems that are reusable without reprocessing.

Similarly to CA/09 H1N1 aerosols, increased protection *in vivo* due to higher filtration efficiency of salt-coated filters compared to bare filters and deactivation of virus on salt-coated filters were observed following exposure to PR/34 H1N1 and VN/04 H5N1 (Fig. 4a,b). This suggests that salt-coated filters prevent virus penetration and destroy virus attached to the filter in a non-specific way. Furthermore, the performance of salt-coated filters was not degraded by storage at 37 °C and 70% RH, demonstrating that salt recrystallization-based filters can ensure protection even under harsh environmental conditions. Notably, for demonstration of the concept of salt-recrystallization based virus deactivation system, NaCl salt was used, which has a critical RH of 75% at 30 °C^35^. However, salts with higher critical RH can be easily used, such as ammonium sulfate, potassium chloride and potassium sulfate, which have critical RH of 80%, 84% and 96.3% at 30 °C, respectively^35^. This suggests that salt-coated filters may be developed for specific environmental conditions.

In conclusion, we demonstrated that the developed salt-recrystallization based filtration system provides high filtration efficiency and successfully deactivates multiple subtypes of adsorbed viruses. Moreover, we have shown that stability of the salt coating is not compromised by high temperature and humidity, which suggests safe use and long-term storage/reuse at such environmental conditions. Although our tests are based on exposure to different types of influenza virus, the significance of these results for personal and public protective measures may be generally extended to enveloped respiratory viruses where infection and transmission can occur by aerosol. Our salt-coated filter unit can promise the development of long-term stable, versatile airborne pathogen negation system, without safety concerns. In fact, the destruction mechanism of viruses solely depends on the simple, yet robust naturally occurring salt recrystallization process, combining the destabilizing effects of salt crystal growth and concentration increase during drying of aerosols. This idea can be easily applied to a wide range of existing technologies to obtain low-cost, universal personal and public means of protection against airborne pathogens, such as masks and air filters in hospitals. Therefore, we believe that salt-recrystallization based virus deactivation system can contribute to global health by providing a more reliable means of preventing transmission and infection of pandemic or epidemic diseases and bioterrorism.

## Methods

### Bare and salt-coated filter samples preparation

The commercial surgical masks had a three-ply structure. The middle layer is the filter media, whereas the inner and outer layers provide support and protect the filter against wear and tear. The metal nose clips and elastic ear loops were removed and circular samples (radius: 3 cm) were cut from the masks. The PP filters (middle layer) were isolated by removing the inner and outer protective layers (bare filters, Filter_bare_). The coating solution was prepared by dissolving sodium chloride (NaCl; Sigma Aldrich, St. Louis, MO) in filtered DI water (0.22 μm pore size; Corning, Tewksbury, MA) under stirring at 400 rpm and 90 °C, followed by the addition of Tween 20 (Fisher Scientific) to a final concentration of 29.03 w/v% of NaCl and 1 v/v% of Tween 20. To obtain the salt-coated filters, the mask bare PP filters were pre-wet to contain approximately 600 μL of coating solution by incubating overnight at room temperature. Any remaining dry areas were removed by applying gentle strokes with tweezers to the filters while immersed in the coating solution. Subsequently, the filters were deposited in the desired volume of coating solution (0, 100, 300, 600, 900 and 1200 μL, of which corresponding membranes are abbreviated as Filter_wet_, Filter_wet+100μL_, Filter_wet+300μL_, Filter_wet+600μL_, Filter_wet+900μL_, and Filter_wet+1200μL_, respectively) on petri dishes (60 × 15 mm; Fisher Scientific) to control the amount of NaCl per unit area and dried in an oven (Isotemp Incubator, Fisher Scientific) at 37 °C for 1 day.

### Influenza virus preparation

Influenza viruses A/California/04/2009 (CA/09, H1N1), A/Puerto Rico/8/34 (PR/34, H1N1) and A/Vietnam/1203/2004 (VN/04, H5N1) were grown in 10-day old embryonated hen eggs, in which H5N1 virus was derived by reverse genetics from HPAI A/Vietnam/1203/2004^36^. Influenza viruses were purified from allantoic fluid using discontinuous sucrose gradient (15%, 30% and 60%) layers following the previously reported procedure^37^.

### Aerosols exposure to filters

For experiments involving aerosols exposure, an aerosol chamber (L × W × H = 145 × 145 × 150 mm; Emka Inc., Middletown, PA) was used (Fig. S11). It has a connection to the vacuum line and a circular aperture in the top wall (diameter: 22 mm) to exactly accommodate the cylindrical part (diameter: 20 mm, height; 20 mm) of the nebulizer unit that is below the aerosol generator (Aeroneb Lab Nebulizer System; Aerogen, Galway, Ireland). Bleach was used as trap between the chamber and the vacuum pump (Welch 2522C-10, 22 L/min; Niles, IL). The filters were placed on top of the chamber aperture and the nebulizer unit was inserted, ensuring the tight seal of the filters against the side of the aperture. 5 μL of virus stock were added to the nebulizer unit, aerosols (VMD 2.5–4 μm from manufacturer specifications) were generated for 30 sec and subsequently the desired vacuum level (3, 10 or 17 kPa) was applied, by manual control, three times in 1 sec cycles. Notably, in the case of bare filters, pressure was only applied for filtration efficiency tests.

For all assays and analysis, suspensions of the filters were prepared as follows, unless otherwise indicated. To reconstitute virus adsorbed onto filters, virus-laden filters were immersed in 400 μL of sterilized DI water for about 5 min, and then removed after vortexing from the suspension. The virus suspension was centrifuged at 19,800 g and 4 °C for 10 min (Centrifuge 5810 R, Eppendorf, Hauppauge, NY), followed by resuspension of pellets in 70 μL of DI water to eliminate any interference from materials in supernatant during assays.

### Filtration efficiency tests

The filters were exposed to the virus aerosols at 3, 10 and 17 kPa and suspensions of the filters were obtained, as described above. The filtration efficiency was calculated as the ratio of the amount of virus (i.e., total proteins measured from the virus) reconstituted from the filter to that from the virus in the exposure aerosols. The concentration of virus in aerosols was determined by generating viral aerosols into a 15 mL centrifuge tube, containing 1 mL of DI water. After vortexing, virus concentrations (i.e., total protein concentration) were measured with bicinchoninic acid assay (BCA protein assay kit; Thermo Fischer scientific, Waltham, IL) with bovine serum albumin as a standard. In the case of virus reconstituted from salt-coated filters, virus-laden filter suspension was replaced with DI water prior to BCA assay.

### *In vivo* infection tests

Lethal infectivity of influenza viruses (CA/09 H1N1) was examined in 8 week old female inbred BALB/c mice (Nara Biotech; Seoul, Korea) by using the intranasal route. For bare and salt-coated filters, 12 mice per group were infected with individual penetration dosage of influenza virus through each filter. The penetration dosage of the virus through the filters (Filter_bare_, Filter_wet_, Filter_wet+600μL_, and Filter_wet+1200μL_) was calculated from the filtration efficiency at 10 kPa (near breathing pressure) using the relationship: penetration dosage = virus dosage in lethal aerosol × penetration efficiency (%)/100, where penetration efficiency (%) = 100 − filtration efficiency (%). To examine the effects of the aerosolization process on the viral infectivity change, two mice groups were infected with a lethal dose of virus before and after aerosol formation, which served as negative control groups. Body weight changes and survival rate of mice were monitored daily for 15 days. Mice with body weight loss greater than 25% were euthanized. All animal protocols were approved by the Kyung Hee University (KHU) Institutional Animal Care and Use Committee (IACUC). All animal experiments and husbandry involved in this work were conducted under the approved protocols and guidelines of KHU IACUC. KHU IACUC operates under National Veterinary Research and Quarantine Service (NVRQS), and animal welfare law and regulations of the WOAH-OIE (World organization for animal health).

To test strain-dependent lethal infection behavior, mice (12 per group) were infected with the penetrated dosage of viral aerosols (PR/34 H1N1 and VN/04 H5N1 viruses) through Filter_wet+600μL_ at 10 kPa. Time-dependent body weight change was monitored in the same manner described above.

### Lung viral titer and lung inflammatory cytokine assays after infection

On day 4 after infection 6 mice of each group were sacrificed for the collection of lung samples. Lung virus titers were measured on six-well plates containing confluent MDCK cell monolayers. Inflammatory cytokines (IFN-γ) were determined using BD OptEIA mouse IFN-γ ELISA kit (BD Biosciences, San Jose, CA) following the manufacturer’s procedure.

### Test of viral infectivity change on filters

To investigate the effects of salt-coating on viral infectivity loss, lethal influenza aerosols were exposed to four different types of filters (Filter_bare_, Filter_wet_, Filter_wet+600μL_, and Filter_wet+1200μL_). Since Filter_bare_ exhibited almost complete penetration upon pressure application, aerosols were exposed to the bare filter in the absence of pressure and samples were carefully handled to prevent mechanical agitation. To measure time-dependent stability change of virus, virus-laden filters were incubated at ambient conditions for 0, 5, 15, and 60 min after aerosol exposure, and suspended in DI water to reconstitute virus at each time point. *In vitro* stability of virus was characterized by measuring hemagglutinin activity (HA) and virus titers at the same concentration as lethal dose^30^. The conformational stability of antigenic proteins was characterized by measuring intrinsic fluorescence using 0.1 mg/mL of virus suspension^38^. To investigate morphological change of virus, lipid stability of viral wall was characterized by nile red fluorescence (Sigma Aldrich), a fluorescent lipid stain, following manufacturer’s protocol^39^. A decrease in fluorescence intensity can be used to examine the level of disintegration of the virus. Both intrinsic and nile red fluorescence were measured by using a fluorimeter (LB 50B; PerkinElmer, Waltham, MA). Intensity changes of fluorescent spectra were compared relative to those of a control from virus stock.

To test infectivity difference observed from *in vitro* findings, *in vivo* study was performed for the virus reconstituted from the filters (Filter_bare_, Filter_wet_, Filter_wet+600μL_, and Filter_wet+1200μL_) after incubation for 60 min at RT (aerosol exposure at 10 kPa, except for Filter_bare_). 12 mice per group were infected with a lethal dose of virus collected from each type of filter. Body weight change and lung virus titers were measured as described above.

### Effects of environmental conditions on the performance of salt-coated filter

Salt-coated filters (Filter_wet_, Filter_wet+600μL_, and Filter_wet+1200μL_) were stored at 37 °C, 70% RH in an incubator (Maru Max; Rcom, Gyeonggi-do, South Korea) for 15 days. Every day, the filters were collected and incubated at ambient conditions for 5 min. At 1-day incubation, filtration efficiency was measured at 10 kPa from Filter_wet+600μL_, followed by *in vivo* infection test. Lethal infectivity between two different filter groups (before and after incubation at 37 °C, 70% RH) was compared by measuring body weight change and survival rate of mice after exposure to lethal CA/09 H1N1 aerosols. XRD analysis was performed to salt-coated filters incubated for 1 and 15 days, and SEM/EDX mapping analysis for 15-day incubated samples.

### Contact angle measurements and imaging of aerosols

The bare and salt-coated filters were fixed with carbon tape (Ted Pella, Inc., Redding, CA) to a metal, flat substrate and 3 μL of DI water were added on the surface of the filters. The contact angles were measured from images collected with an optical microscope (10× lens, Motic SMZ-140; Motic, Richmond, Canada) at RT. Images of aerosols on filter fibers were obtained using a dispersive Raman microscope (Nicolet Almega XR; Fisher Scientific).

### Aerosol drying time on filters

The bare and salt-coated filters were fixed with carbon tape to a metal, flat substrate and exposed to aerosols generated from 5 μL of Sulforhodamine B Dye solution (1 mM, Sigma-Aldrich). Aerosol drying time was determined with timer by observation with optical microscope.

### Electron microscopy analysis

For virus stability tests, bare and salt-coated filters were exposed to CA/09 H1N1 aerosols and, after 5 and 60 min incubation, virus was recovered by suspension of the filters, as described above. To study the effects of the coating formulation during aerosol drying independently from crystal growth, bare and salt-coated filters were immersed in DI water and removed after 60 min. Subsequently, virus was incubated in the obtained suspension for 60 min. Additionally, the virus suspension was centrifuged at 19,800 g and 4 °C for 10 min to collect the samples and suspend them in DI water. For TEM analysis (200 kV, JEOL JEM 2100; JEOL, Peabody, MA), samples were deposited on copper grid (Electron Microscopy Sciences, Hatfield, PA) and negatively stained with solution comprised of phosphotungstic acid hydrate (1.5 w/v%, pH = 7.0; Sigma-Aldrich, Oakville, Canada).

To identify the morphology of salt-coated filters and recrystallized salts, SEM/EDX analysis was performed for bare and salt-coated filters after coating with 10 nm thick gold layer. Scanning electron microscopy analysis (Hitachi S-3000N; Hitachi, Toronto, Canada) was operated in secondary electron mode at 20 kV and EDX analysis was obtained with EDX detector (Oxford Instruments, Concord, MA).

### XRD analysis

To confirm the formation of crystalline NaCl coating during drying process and its stability during storage at 37 °C and 70% RH, XRD analysis (BRU-1098; Bruker, Billerica, MA) was performed at different coating conditions. Filters (1 × 1 cm) were mounted on a slide glass for XRD analysis (θ–2θ mode) using a CuKα radiation.

### Statistical analysis

To compare multiple conditions, Student’s t-test, One-way analysis of variance (ANOVA), and general linear model were used (Minitab release 14; Minitab, State College, PA). *P* value of less than 0.05 was considered to be significant.

## Additional Information

**How to cite this article**: Quan, F.-S. *et al*. Universal and reusable virus deactivation system for respiratory protection. *Sci. Rep.* **7**, 39956; doi: 10.1038/srep39956 (2017).

**Publisher's note:** Springer Nature remains neutral with regard to jurisdictional claims in published maps and institutional affiliations.

## Supplementary Material

Supplementary Information
