# Supplementary material for: Universal and reusable virus deactivation system for respiratory protection
Source: Sci Rep. 2017 Jan 4;7:39956. doi: 10.1038/srep39956 (PMC5209731; doi:10.1038/srep39956)
Supplement: Supplementary Information [file srep39956-s1.pdf]

Universal and reusable virus deactivation system for respiratory protection

Fu-Shi Quan[1,†], Ilaria Rubino[2,†], Su-Hwa Lee[3], Brendan Koch[2], and Hyo-Jick Choi[2,*]

[1]Department of Medical Zoology, Kyung Hee University School of Medicine, Seoul, 130-701, Korea.

[2]Department of Chemical and Materials Engineering, University of Alberta, Edmonton, AB T6G 1H9, Canada.

[3]Department of Biomedical Science, Graduate School, Kyung Hee University, Seoul, 130-701, Korea.

[*] Correspondence to: hyojick@ualberta.ca (HJC).

[†] These authors contributed equally to this work.

Supplementary figures

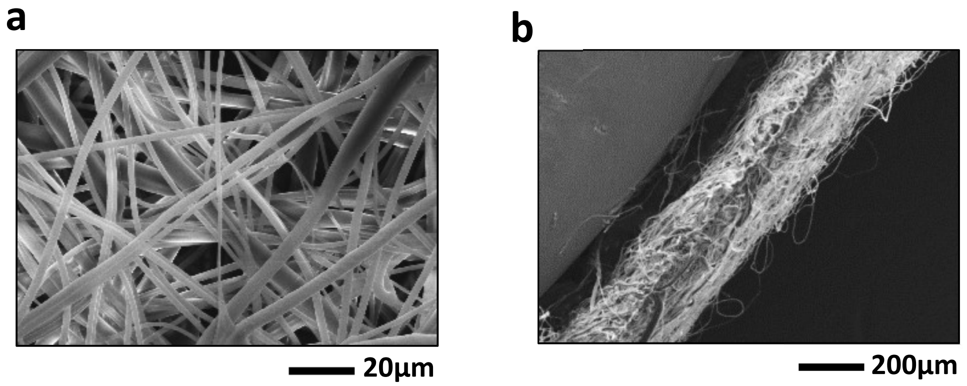

Supplementary Fig. S1

SEM micrographs of PP filter of bare surgical mask ($Filter_{bare}$). (**a**) Top view and (**b**) cross-sectional view.

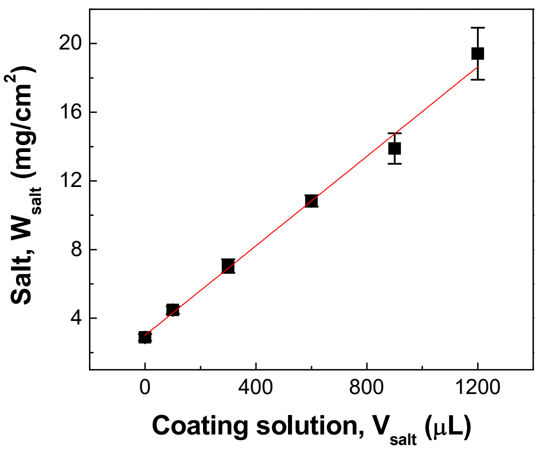

Supplementary Fig. S2

Plot showing the relationship between the volume of coating solution used for drying of pre-wet filters (V_{salt}) and amount of coated salts (W_{salt}) (n = 7, mean ± SD).

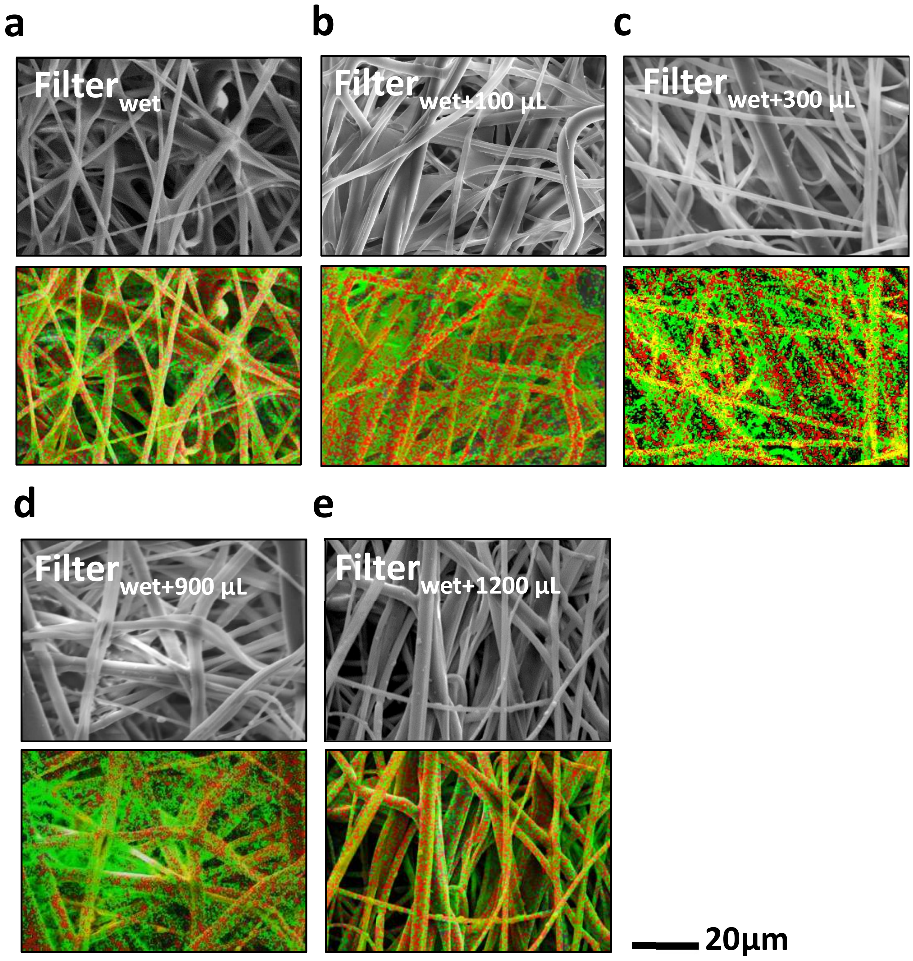

Supplementary Fig. S3

Representative SEM/EDX mapping images of salt-coated filters. (**a**) $Filter_{wet}$, (**b**) $Filter_{wet+100μL}$, (**c**) $Filter_{wet+300μL}$, (**d**) $Filter_{wet+900μL}$, and (**e**) $Filter_{wet+1200μL}$ (top: SEM, bottom: EDX mapping).

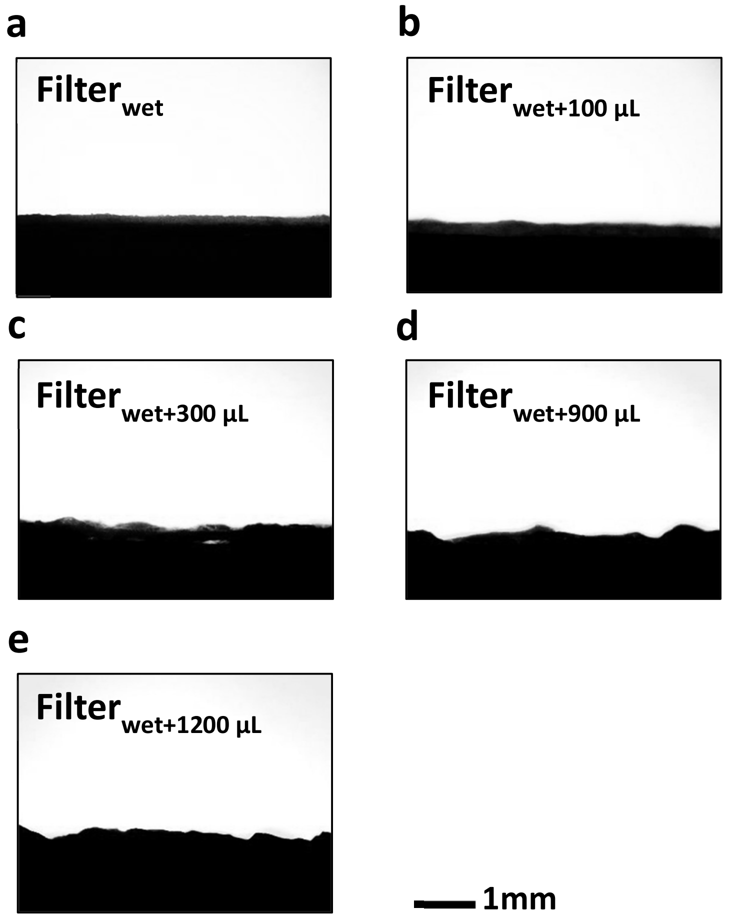

Supplementary Fig. S4

Optical microscope images of cross-sectional view of salt-coated filters after applying a drop of DI water (3 μL) for contact angle measurements. All of salt-coated filters exhibited complete wetting (n = 10).

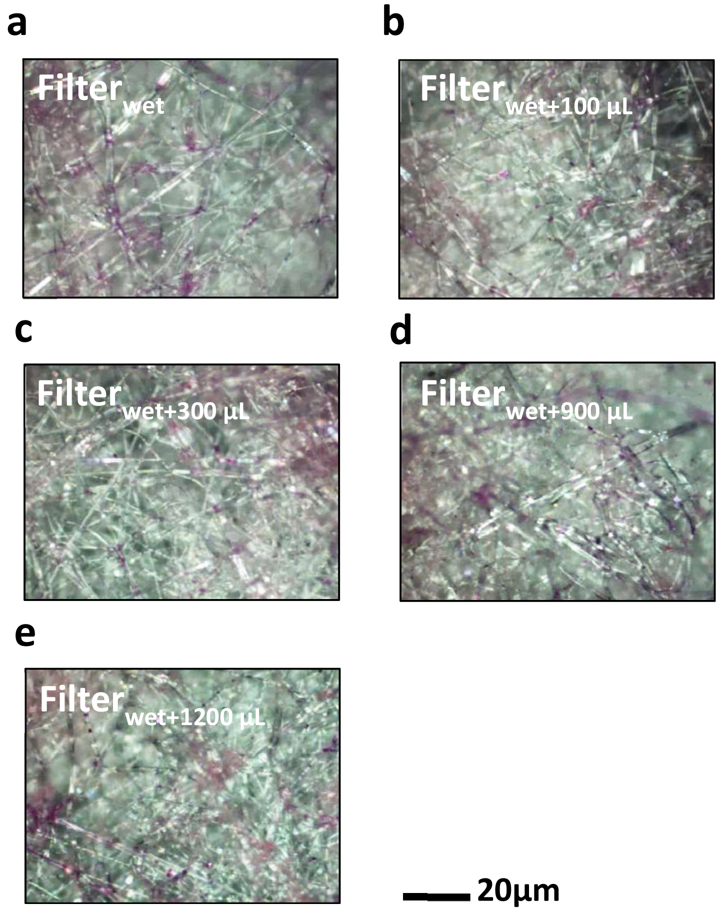

Supplementary Fig. S5

Optical microscope images of top view of salt-coated filters right after exposure to aerosols. All of salt-coated filters exhibited complete wetting (n = 10).

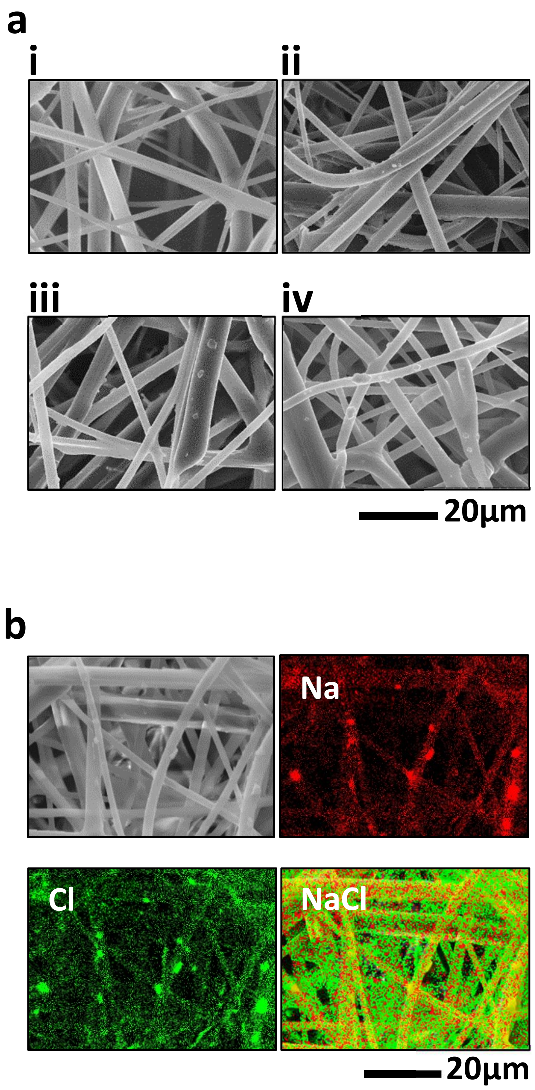

Supplementary Fig. S6

(**a**) SEM images of filters incubated for 60 min after exposure to influenza virus (i: $Filter_{bare}$, ii: $Filter_{wet}$, iii: $Filter_{wet+600\mu L}$, iv: $Filter_{wet+1200\mu L}$) showing the micron-sized NaCl phase on salt-coated filters upon drying of aerosols. (**b**) SEM/EDX mapping images of $Filter_{wet+600\mu L}$ exposed to aerosols (Na: red, Cl: green). Micron-sized structure on the filter is identified as NaCl phase due to NaCl salt recrystallization.

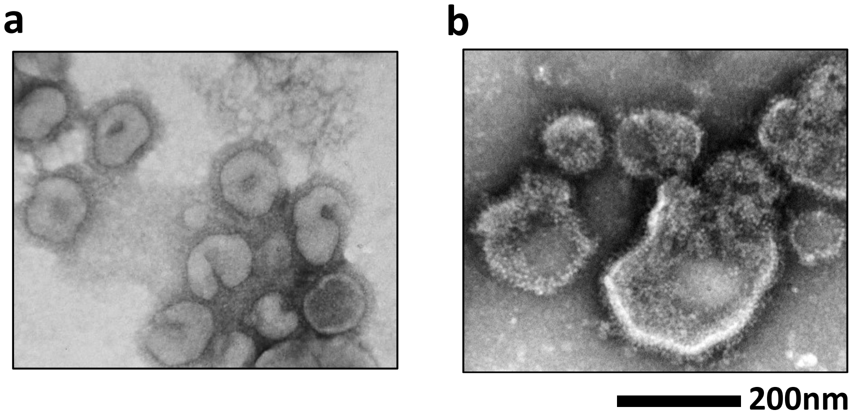

Supplementary Fig. S7

Negative-stain TEM micrographs of A/CA/04/2009 (H1N1) influenza virus incubated for 1 hr in solution obtained from suspension of (**a**) $Filter_{bare}$ and (**b**) $Filter_{wet+600\mu L}$ showing the effects of high salt/surfactant concentration and osmotic pressure on virus morphology.

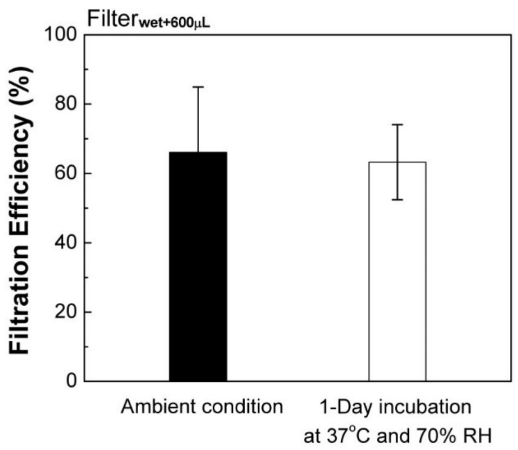

Supplementary Fig. S8

Filtration efficiency of $Filter_{wet+600\mu L}$ before and after 1 day incubation at 37°C and 70% RH (*n* = 12, mean ± SD).

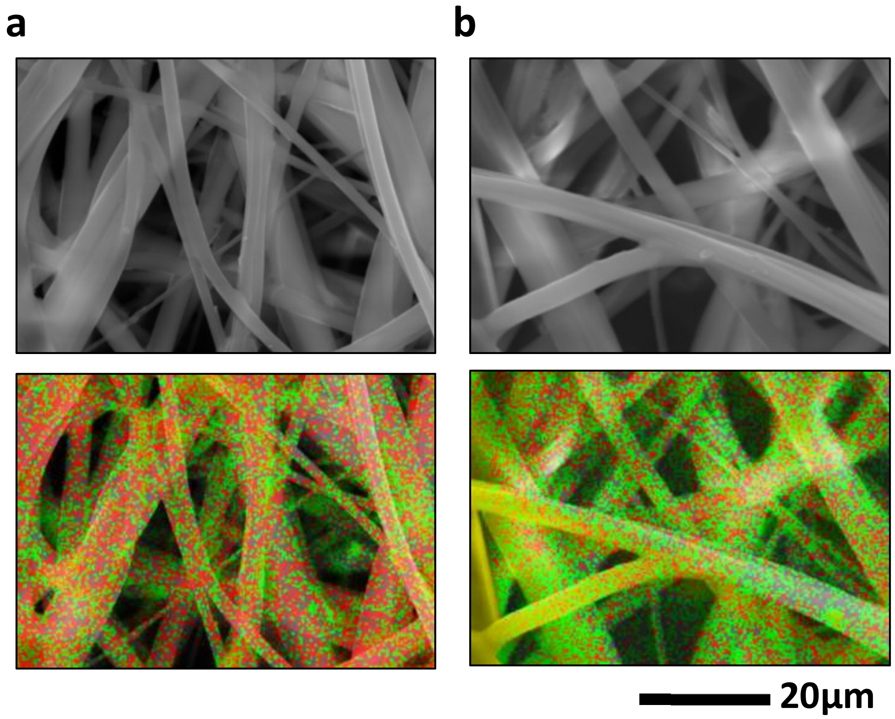

Supplementary Fig. S9

SEM (top)/EDX mapping (bottom) images of (**a**) $Filter_{wet}$ and (**b**) $Filter_{wet+1200\mu L}$ after incubation for 15 days at 37°C and 70% RH.

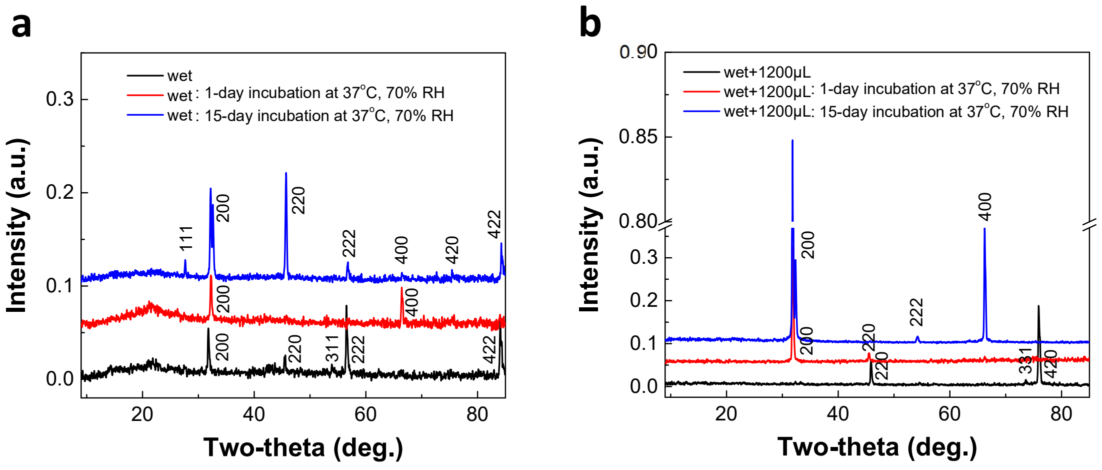

Supplementary Fig. S10

XRD spectra of (**a**) Filter$_{wet}$ and (**b**) Filter$_{wet+1200\mu L}$ before and after incubation at 37°C and 70% for 1 day and 15 days.

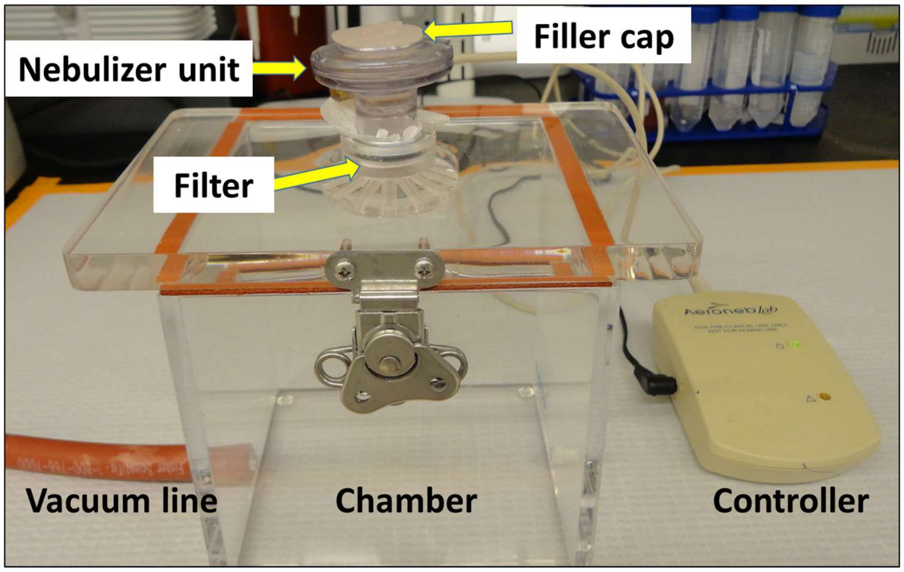

Supplementary Fig. S11
Experimental setup used to measure filter performance.
